# Mapping Protein Interactions between Dengue Virus and Its Human and Insect Hosts

**DOI:** 10.1371/journal.pntd.0000954

**Published:** 2011-02-15

**Authors:** Janet M. Doolittle, Shawn M. Gomez

**Affiliations:** 1 Curriculum in Bioinformatics and Computational Biology, University of North Carolina at Chapel Hill, Chapel Hill, North Carolina, United States of America; 2 Joint Department of Biomedical Engineering, University of North Carolina School of Medicine, Chapel Hill, North Carolina, United States of America; 3 Department of Computer Science, University of North Carolina at Chapel Hill, Chapel Hill, North Carolina, United States of America; 4 Department of Pharmacology, University of North Carolina School of Medicine, Chapel Hill, North Carolina, United States of America; National Yang-Ming University, Taiwan

## Abstract

**Background:**

Dengue fever is an increasingly significant arthropod-borne viral disease, with at least 50 million cases per year worldwide. As with other viral pathogens, dengue virus is dependent on its host to perform the bulk of functions necessary for viral survival and replication. To be successful, dengue must manipulate host cell biological processes towards its own ends, while avoiding elimination by the immune system. Protein-protein interactions between the virus and its host are one avenue through which dengue can connect and exploit these host cellular pathways and processes.

**Methodology/Principal Findings:**

We implemented a computational approach to predict interactions between Dengue virus (DENV) and both of its hosts, *Homo sapiens* and the insect vector *Aedes aegypti*. Our approach is based on structural similarity between DENV and host proteins and incorporates knowledge from the literature to further support a subset of the predictions. We predict over 4,000 interactions between DENV and humans, as well as 176 interactions between DENV and *A. aegypti*. Additional filtering based on shared Gene Ontology cellular component annotation reduced the number of predictions to approximately 2,000 for humans and 18 for *A. aegypti*. Of 19 experimentally validated interactions between DENV and humans extracted from the literature, this method was able to predict nearly half (9). Additional predictions suggest specific interactions between virus and host proteins relevant to interferon signaling, transcriptional regulation, stress, and the unfolded protein response.

**Conclusions/Significance:**

Dengue virus manipulates cellular processes to its advantage through specific interactions with the host's protein interaction network. The interaction networks presented here provide a set of hypothesis for further experimental investigation into the DENV life cycle as well as potential therapeutic targets.

## Author Summary

Dengue virus (DENV) represents a major disease burden in tropical and subtropical regions of the world, and has shown an increase in the number of cases in recent years. DENV is transmitted to humans through the bite of an infected mosquito, typically *Aedes aegypti*, after which it begins the infection and replication lifecycle within human cells. To perform the molecular functions required for invasion, replication, and spread of the virus, proteins encoded by DENV must interact with and alter the behavior of protein networks in both of these hosts. In this work, we used a computational method based on protein structures to predict interactions between DENV and its human and insect hosts. We predict numerous interactions, with many involved in known cell death, stress, and immune system pathways. Further investigation of these predicted protein-protein interactions should provide targets to combat the clinical manifestations of this disease in humans as well as points of intervention focused within the mosquito vector.

## Introduction

With over 50 million cases per year, dengue virus (DENV) is a significant and growing threat to world-wide human health. Wide-spread among tropical and sub-tropical regions, this NIAID Category A pathogen consists of four serotypes, DENV1 to DENV4, and is a member of the family *Flaviviridae* [1]. DENV causes a range of diseases in humans, from the mild Dengue Fever (DF) to the more deadly Dengue Hemorrhagic Fever (DHF) and Dengue Shock Syndrome (DSS). While both the average number of cases reported to the WHO as well as the number of countries reporting cases of DENV have increased dramatically in the past five decades, relatively little is known about this important tropical pathogen that still lacks a vaccine, specific drug treatment, and relevant animal model [2].

An arbovirus, DENV is carried and spread to humans by the primary mosquito vector *Aedes aegypti* and to a lesser extent *Aedes albopictus*. Thus, DENV displays the remarkable capability to survive and replicate in two very different host organisms; accomplished by a genome encoding a mere 10 proteins [3]. To be successful, DENV must be able to manipulate each of its hosts at a molecular level. This manipulation must be accomplished, in part, through specific protein-protein interactions that allow the virus to bend existing host cellular systems to the purpose of furthering the viral lifecycle. However, understanding this host-pathogen system is particularly difficult given the complexities of host-virus dynamics as well as the lack of a useful animal model system. In light of these challenges, computational approaches provide an important tool in studies of host-pathogen systems. In particular, computational approaches for predicting host-pathogen protein interactions provide opportunities for identifying specific targets for further experimental work, understanding system behavior, and determining plausible therapeutic candidates.

While common within model organism species such as *S. cerevisiae*, prediction of protein interactions between species are rare, and are especially so for host-pathogen interactions. Recent computational work has considered interactions between *P. falciparum* and human proteins based on interactions between orthologous eukaryotic proteins or using statistics about protein domains involved in within-species interactions [4], [5]. For the HIV-human system, Tastan *et al.* used a data mining approach to predict host-virus interactions based on human protein features and knowledge of existing protein interactions [6]. Also focused on HIV, Evans and colleagues used short sequence motifs conserved in both HIV and humans as the basis for interaction predictions [7].

Protein structure information can also be used as a basis for protein interaction prediction [8]–[10]. Here, given a set of proteins with defined structures and known interactions, interactions can be mapped to another set of proteins possessing similar structures. This has been applied to HIV-human interactions as well as to non-viral pathogens for a number of tropical diseases [11], [12]. Unfortunately, despite their potential value, such computational structure approaches have not been widely applied to the problem of predicting host-pathogen interactions. In particular, we are not aware of any studies focused on computational large-scale prediction of protein-protein interactions between DENV and humans and know of only one recent study related to *Aedes* [13].

**Figure 1:**
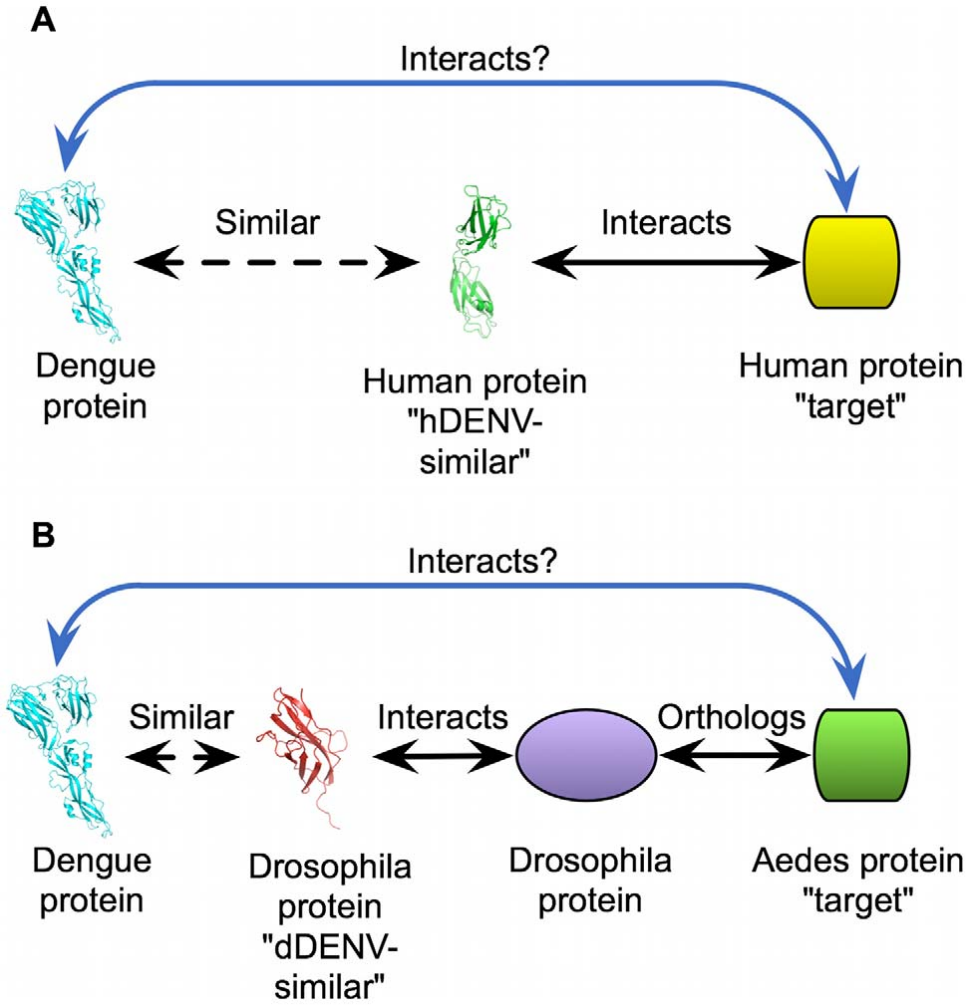
Diagram of approach. (A) Predictions for the human host. Interactions between DENV proteins and human targets is predicted on the basis of structural similarity between the DENV protein and an hDENV-similar protein, and the hDENV-similar protein's known interaction with the human target. (B) Predictions for the insect host are made in a similar manner as (A), except for the additional step of finding orthologs of the *D. melanogaster* target proteins in the real host of interest, *A. aegypti*.

Here, we establish a network of predicted interactions between DENV proteins and proteins from its human and insect hosts. These predictions are based on protein structural similarity, where we first determine structural similarities between pathogen and host proteins using an established method for comparing 3D structures. We refer to host proteins having a region of high structural similarity to a DENV protein as “DENV-similar.” Next, we identify known intra-species interactions for these DENV-similar proteins, and refer to the one or more host proteins that they interact with as “targets.” We then assume that the similar structural features seen between DENV proteins and their host DENV-similar counterparts allow the DENV protein to participate in the same interactions as DENV-similar proteins; joining the host protein network at these points (Figure 1). We prioritize the interaction map using data from recent RNAi screens, to create a smaller network of interactions having the greatest potential to be correct. These predictions include numerous novel interactions with potential functional relevance and we highlight predictions relevant to stress, the Unfolded Protein Response (UPR) as well as interferon pathways. This computational network approach provides an additional tool for the investigation of poorly-characterized host-pathogen systems such as DENV, as well as helping to identify potential targets in both hosts that may be used in future DENV vaccination, treatment, and control efforts.

## Methods

### Data Sources

Structures of DENV2 proteins were taken from the PDB (downloaded on Dec. 9, 2009), and any DENV2 protein without a known structure was modeled using I-TASSER [14], [15]. The protein sequences used to create I-TASSER models were Entrez Protein 159024813, 159024814, 159024817, and 159024819. Default settings were used, with no restraints nor selection/exclusion of any templates. Each of the structures for DENV proteins was run on the DaliLite v. 3 webserver [16], [17]. HPRD Release 7 was used to obtain known human protein-protein interactions, while known *D. melanogaster* interactions were taken from DroID v5.0 and IntAct [18]–[20]. The *A. aegypti* orthologs of *D. melanogaster* proteins were determined using the Inparanoid ortholog annotation for the *D. melanogaster* genes in FlyBase v. FB2009 10 [21], [22]. The literature sources and various databases used each have their own system of identifiers. PDB codes obtained from Dali were mapped to their corresponding taxonomy and Uniprot accessions using data from the SIFTS initiative, which aims to ease the integration of data from multiple databases (http://www.ebi.ac.uk/msd/sifts/) [14], [23]. Other identifier mappings were carried out using DAVID Gene ID Conversion or Uniprot ID mapping (DAVID 6.7, Uniprot Release 15.14) [24]–[26]. Network diagrams were created in Cytoscape [27]. Images of protein structures were created in MacPyMol [28].

### Determination of Structural Similarity between DENV and Host Proteins

We investigated protein mimicry using structural similarities from Dali. Dali compares the 3D structural coordinates of two PDB entries by alignment of alpha carbon distance matrices, allowing for differences in domain order, and produces a structural similarity score [14], [16], [17]. For this study, we ran each of the DENV2 protein structures, both known and predicted, through the DaliLite webserver, which searched against the entire PDB for structurally similar proteins, with a z score above 2.0. Default settings of a score cutoff of 40 bits and sequence overlap cutoff of 50% were used. We then took from these results only those structures that were from the species *H. sapiens* and *D. melanogaster*. We refer to these human proteins as hDENV-similar proteins and the fly proteins as dDENV-similar proteins.

### Interaction Prediction

To predict which human proteins may interact with DENV2 proteins, we sought those target human proteins that interact with the hDENV-similar proteins during cellular processes. To this end, we determined known interactions between hDENV-similar proteins and target human proteins, using data from the Human Protein Reference Database (HPRD) database, which contains literature curated interactions between pairs of human proteins [18]. For each hDENV-similar protein, we predicted that the target proteins which are known to interact with the hDENV-similar protein might also interact with that DENV protein.

A similar process was used to predict interactions between DENV2 proteins and *A. aegypti* proteins, but with the added step of finding orthologs between *A. aegypti* and *D. melanogaster* proteins. Known interactions between the dDENV-similar proteins and other *D. melanogaster* proteins were taken from DroID, using a cutoff confidence value of 0.4, and IntAct [19], [20]. Then, orthologs of the *D. melanogaster* proteins were found for *A. aegypti* using FlyBase [22]. We then made the prediction that the *A. aegypti* target protein interacts with the DENV protein.

### GO Term Enrichment

The Gene Ontology (GO) provides a system of terms to consistently describe and annotate gene products [29]. GO term enrichment was performed using the DAVID Functional Annotation Chart tool [24], [25]. The GO is organized as a tree structure, with terms becoming more specific as distance from the root increases. Therefore, to avoid very general and uninformative GO terms, we used only GO level 4 terms. The p-values were corrected for multiple testing using the Bonferroni procedure and 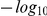 transformed.

### Validation of Predictions

Since there may be multiple PDB structures present in Dali to represent the same protein, there was some redundancy in the interaction predictions. In some cases, multiple PDB structures for the same DENV protein were found to be similar to multiple PDB structures for an DENV-similar protein, leading to the same interaction predictions. Therefore, the predictions were counted as unique pairs of human Uniprot accessions and DENV protein names. In addition, for ease of viewing the predicted interactome, each node representing an DENV protein is labeled with the protein name while each human protein is represented by Entrez GeneID.

Support for the predicted interactions was obtained from literature. As few interactions between DENV and humans are known, we looked within the literature to see if any of them were predicted by our method [30]–[33]. In addition, recent studies using siRNA screens have found proteins that may play some role, either facilitatory or inhibitory in DENV infection, in both humans and *D. melanogaster* [34], [35]. *A. aegypti* orthologs of these host factors were recently curated by Guo et al. [26]. We checked for the presence of these human host factors or mosquito orthologs among our predictions. Although it is not known if these proteins act through direct protein-protein interactions with DENV or indirect mechanisms, their involvement in DENV infection provides functional support for a possible interaction and gives them higher priority for further testing.

### GO Cellular Components Filter

GO annotations for the human and *A. aegypti* target proteins were obtained through DAVID 6.7 [24], [25]. However, since DAVID assigns all DENV proteins the same GO terms, GO annotation for the DENV proteins was obtained using the GOanna webserver, provided through AgBase v. 2.00 [36]. This tool assigns GO terms to the input sequences by transitively assigning the GO terms of similar, already annotated sequences identified by BLAST. The most significant BLAST hits for the DENV protein sequences were DENV polyprotein sequences. However, there were multiple polyprotein sequences, each with their own annotations. The input sequences matched more significantly to some polyproteins than to others, and were therefore assigned different GO terms based on sequence similarity. The predicted interactions were filtered so that only those predictions for which the DENV protein and host protein shared at least one GO cellular component term were retained.

### Predictions Using Mosquito Interactome

Guo et al. recently generated a first draft of the mosquito interactome [13]. Because their interactome was based on the three model organisms *A. aegypti*, *C. elegans*, and *S. cerevisiae*, we found proteins from all three of these species that show structural similarity with DENV2 using the Dali server [14], [16]. The *A. aegypti* orthologs of *C. elegans* and *S. cerevisiae* proteins were determined using InParanoid, and the *D. melanogaster* orthologs were taken from the InParanoid ortholog annotation for the *D. melanogaster* genes in FlyBase v. FB2009 10 [21], [22]. Then, the interactions with these orthologs taken from the mosquito interactome were used to map predicted interactions between DENV2 and mosquito target proteins. For the GO term enrichment, we used only GO terms from DAVID's GO fat set, to eliminate non-specific terms with many children.

### Determining Orthologous Targets

The genome-wide set of orthologs between human and *A. aegypti* was downloaded from InParanoid 7.0 Refworks:161. Since InParanoid lists human proteins by their Ensembl Protein IDs, mappings to Uniprot accessions were downloaded from Ensembl 57 using BioMart Refworks:192. Orthologous human and *A. aegypti* targets that were predicted to interact with the same DENV2 protein were identified.

## Results and Discussion

### Identification of Dengue-Similar Host Proteins

To develop a network of interactions between DENV and its hosts, *H. sapiens* and *A. aegypti*, we employed a method we developed previously in the prediction of protein interactions between HIV and human [12] (see Methods for further details). First, we obtained 3D structures for the DENV proteins, from two sources. Experimentally determined structures were taken from the PDB and consist of 31 PDB entries representing the DENV2 proteins E, pr peptide, prM, C, NS2B, NS3, and NS5 [37]. Since there are no experimentally determined structures for NS1, NS2A, NS4A, and NS4B, we used the I-TASSER server to predict the structure of these proteins [15]. In this way, we investigated possible interactions for every DENV protein.

To determine structurally similar host proteins, we used DaliLite to compare DENV structures against every other structure in the PDB [16], [17], [37]. We considered only significant structural matches with proteins from DENV's hosts. We found 300 human proteins with similarity to a DENV protein (hDENV-similar). However, we found no similarities between DENV proteins and *A. aegypti* proteins. This is not surprising, given there are currently only 17 structures from *A. aegypti* in the PDB. Therefore, we looked for similarities between DENV proteins and the fly, *Drosophila melanogaster*, and found 15 proteins with structural similarity to DENV, which were then used as dDENV-similar proteins in downstream analyses.

### Known DENV-Host Interactions

A particular challenge in host-pathogen studies is the general lack of interaction data. HIV is perhaps the most well-characterized virus in this regard, with over 800 direct interactions documented in NCBI's HIV-Human protein interaction database (over 2500 interactions if indirect interactions are included) [38]. In contrast, a recent compilation of host-pathogen interactions from public databases describes a total of 3 DENV-human interactions [39].

Through a more comprehensive search of the literature, we have found 20 documented interactions between DENV and human proteins (Table 1). Almost half of the documented protein interactions involve E protein and a receptor on the cell surface. Two of these, CD14 and HSPA5, have been shown to function as DENV receptors, although their binary interaction with E protein was not explicitly demonstrated; it may be that some other protein in complex with these receptors is the direct interaction partner of E protein [40], [41]. Furthermore, there is evidence that DENV receptor usage may be virus strain and cell type dependent [42]. Indeed, RPSA has been shown to be a DENV receptor, suggesting an interaction with E, but only for DENV1 [43]. Because our predictions were focused on DENV2, this interaction was not considered for our predictions, but was included in Table 1. Interactions not shown to be specific for a different serotype were included in our list of true positive interactions. Therefore, a total of 19 protein interactions were considered as known host-pathogen interactions between DENV2 and human.

**Table 1:** Known interactions between *H. sapiens* and DENV.

Human	DENV	Serotype	System	Reference
UBE2I	E	2	*in vitro* †	[32]
HSP90AA1	E	2	U937, SK-SY5Y, monocyte	[33]
HSPA4	E	2	U937, SK-SY5Y, monocyte	[33]
HSPA5	E	2	HepG2,Vero	[41], [55]
CANX	E	2	Vero	[55]
CALR	E	2	Vero	[55]
CD14	E	2	primary monocytes/macrophages	[40]
CD209	E	1‡	BHK†	[48]
RPSA	E	1*	HepG2	[43]
DAXX	C	2	HepG2	[49]
HNRNPK	C	2	293T†	[30]
HNRNPC	NS1	2	HEK 293T	[50]
CLU	NS1	2	plasma, 293T, Vero	[51]
STAT3	NS1	2	BHK	[52]
NRBP1	NS3	2	BHK†	[31]
SSB	NS3	4	U937	[47]
SSB	NS5	4	U937	[47]
TJP1	NS5	2	epithelial cells	[54]
STAT2	NS5	2	293T	[80]
PTBP1	NS4A	2	Huh-7	[53]

Experimentally determined nteractions are listed, with the serotype and system that they were demonstrated in.

**†:** Interaction suggested in one or more (additional) cell lines by functional assay.

**‡:** Interaction suggested for other serotypes by functional assay.

*Interaction shown to be specific for this serotype.

We are currently unaware of any well-characterized protein-protein interactions between DENV and *A. aegypti*. However, in the C6/36 cell line from *Aedes albopictus*, tubulin is believed to interact with DENV2 E protein [44]. In addition, one protein, likely to be HSP90, has been put forward as a putative receptor for DENV2 in *A. aegypti*, having been shown to bind to the E protein [45]. However, its identity has not been conclusively demonstrated. In addition, mosquito La auto-antigen is known to interact with the 3′ end of DENV RNA and may play some role in RNA synthesis [46]. Human La auto-antigen (SSB) is also known to interact with the ends of the viral RNA, as well as NS3 and NS5 [47]. If the functions of the mosquito and human La proteins in DENV infection are similarly conserved, mosquito La may interact with NS3 and NS5 as well, although this has not been shown. It is likely that some of the protein interactions which enable DENV to manipulate the cellular pathways of two hosts are conserved between the species.

### Prediction of Protein Interactions

After determining which host proteins are structurally similar to DENV proteins, we inquired into the known protein-protein interactions that each DENV-similar protein participates in. For the hDENV-similar set, we obtained known human protein interactions from the Human Protein Reference Database (HPRD), which consists of over 37,000 interactions found in the literature [18]. We predicted that the DENV proteins could interact with the partners of their corresponding hDENV-similar proteins, under the hypothesis that proteins with highly-similar structures are likely to be involved in similar protein interactions (Figure 1A). We predicted 4,273 potential host-pathogen interactions, involving 2,321 different human proteins (Table 2). Of the 19 known protein-protein interactions between DENV and human, 9 are present among our predictions [30]–[33], [40], [41], [47]–[55]. This method may not predict all interactions, for example those mediated by sequence motifs rather than structural features. A table of all DENV-human protein interaction predictions is provided in Table S1.

**Table 2:** Interaction predictions summary.

	Human	Human CC	Insect	Insect CC
**Dengue-similar**	300	254	15	6
**Targets**	2,321	1,099	158	12
**Known Predictions**	9	7	NA	NA
**Host Factor Predictions**	48	20	12	1
**Total Predictions**	4,273	2,073	176	17

Counts are given for the predictions made between DENV and both the human and *A. aegypti* hosts, both before and after CC filtering. Known Predictions for *A. aegypti* is listed as NA because we are aware of no known protein-protein interactions between DENV and *A. aegypti*.

For the dDENV-similar proteins, we used the interactions curated in IntAct for *D. melanogaster*, as well as potential *D. melanogaster* interactions suggested by the yeast-2-hybrid data sets in DroID [19], [20]. However, rather than making direct predictions using these interactions, as we did for human proteins, we determined orthologs of the *D. melanogaster* proteins in *A. aegypti*, since this is the true host of DENV. We then predict that the *A. aegypti* ortholog of a *D. melanogaster* protein that interacts with a dDENV-similar protein may also interact with the corresponding DENV protein (Figure 1B). As a result of this procedure, we predict that 158 *A. aegypti* proteins participate in 176 interactions with DENV proteins (Table 2). We note that this method did not predict interactions between E and mosquito tubulin, HSP90, or La, which have been suggested as possible interactions [45]–[47]. However, 12 of the predictions involved orthologs of proteins involved in DENV2 infection in humans or fly. A table of all DENV-*Aedes* protein interaction predictions is provided in Table S2.

### Assessment of Predictions

#### GO term enrichment

**Figure 2:**
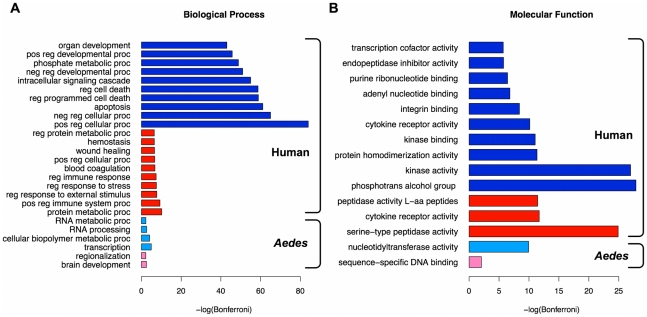
GO term enrichment of host proteins. (A) Enriched GO biological process terms. (B) Enriched GO molecular function terms. Blue bars represent terms enriched among human target proteins, red is terms enriched among hDENV-similar proteins, light blue is terms for *A. aegypti* targets, and pink is for terms from dDENV-similar proteins. When more than ten terms were enriched for a set of proteins, only the ten most significant terms are shown. Bonferroni corrected p-values were transformed by 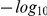. The following abbreviations are used: “reg” is “regulation of,” “pos” is “positive,” “neg” is “negative,” “proc” is “process,” “peptidase activity L-aa peptides” is “peptidase activity acting on L-amino acid peptides,” and “phosphotrans alcohol group” is “phosphotransferase activity alcohol group as acceptor.” Brackets delineate the two host species.

Due to the sparseness of known interactions to which we can compare and evaluate our predictions, we examined the functional roles of host proteins for patterns relevant to DENV infection. To this end, we determined the amount of GO term enrichment among the DENV-similar and target proteins for each host. We find that many of the most significantly enriched terms among these sets of proteins are for processes or functions known to be important for DENV infection (Figure 2). Our results are also consistent with a study of altered protein expression during DENV infection in which several of the proteins identified have functions related to the GO terms RNA processing, transcription, or regulation of stress response, which were enriched in our predictions [56].

For human target proteins (dark blue bars in Figure 2A), processes involving signaling, cell death and apoptosis, and positive or negative regulation terms are much more frequent than would be expected if chosen at random. These processes are in agreement with processes and pathways that are known to be altered during the course of infection. Human proteins with structural similarities to DENV (red bars) are enriched for terms describing blood coagulation and hemostasis, indicating that DENV proteins appear to have structural similarities that mimic human proteins involved in the pathways controlling the cessation of bleeding. One of the defining symptoms of DHF is hemorrhage, the pathogenesis of which has been shown to include abnormalities in levels of cytokines, complement components, and coagulation factors [57], [58]. DENV proteins show structural similarity with proteins from all three of these categories. In fact, DENV mimicry of clotting factors has already been observed: antibodies against the DENV2 protein NS1 have been shown to cross-react with clotting factors and integrins on thrombocytes and endothelial cells [59]. Kinase, cytokine, phosphotransferase, and peptidase functional roles are also enriched in hDENV-similar and human target proteins.

The most enriched biological processes for DENV-*A. aegypti* within our interaction predictions appear to revolve around RNA processing and transcription-related processes. This is observed in enriched functions which include “nucleotidyltransferase activity” and “sequence-specific DNA binding” GO terms (Figure 2B). Gene expression analyses within *A. aegypti* in response to DENV infection have shown changes in several hundred genes across a range of functions, with immune and transcriptional processes being highly represented [60]. We note that one protein with mRNA processing activity, AAEL013723-PA or polypyrimidine tract binding protein, is an ortholog of human PTBP1, which is known to interact with NS4A [53]. However, we predict AAEL013723-PA interacts with NS2A in *A. aegypti* and make no predictions about PTBP1 interacting with DENV in humans (Table S2). In addition, there are several lines of evidence suggesting that *A. aegypti* may use the RNAi pathway as a defense against infection by DENV and variations in the RNAi pathway in both the host and viral strains may contribute to differences in the efficiency of infection [61].

#### Literature filtering

Our prediction of host-DENV interactions using protein structural similarity allows us to generate a list of candidate interactions with potentially significant functional relevance, forming a possible basis for further experimental and computational studies of this system. Given the large number of predictions as well as the sparsity of known interaction information, we wished to incorporate additional data to help refine these predictions into a “increased-confidence” set of interactions. To do this, we incorporated functional information from recent literature. Specifically, two siRNA screens have recently examined the roles of host proteins in DENV infection [34], [35]. Sessions *et al.* performed a genome-wide siRNA screen for *D. melanogaster* proteins whose depletion affected the ability of DENV2 to infect the host cells. For those insect host factors with human homologues, they verified 55 as human host factors, also using siRNA [34]. In addition, Krishnan *et al.* identified 123 human host factors for DENV in a study primarily searching for West Nile Virus host factors, but also testing these proteins in DENV infection [35]. In total, these two studies implicated 173 human proteins and 116 *D. melanogaster* proteins as playing a role in DENV infection. We note that similar studies in HIV together have revealed nearly 1000 such human host factors, and thus it is unlikely that these studies have identified all such factors in DENV hosts.

**Figure 3:**
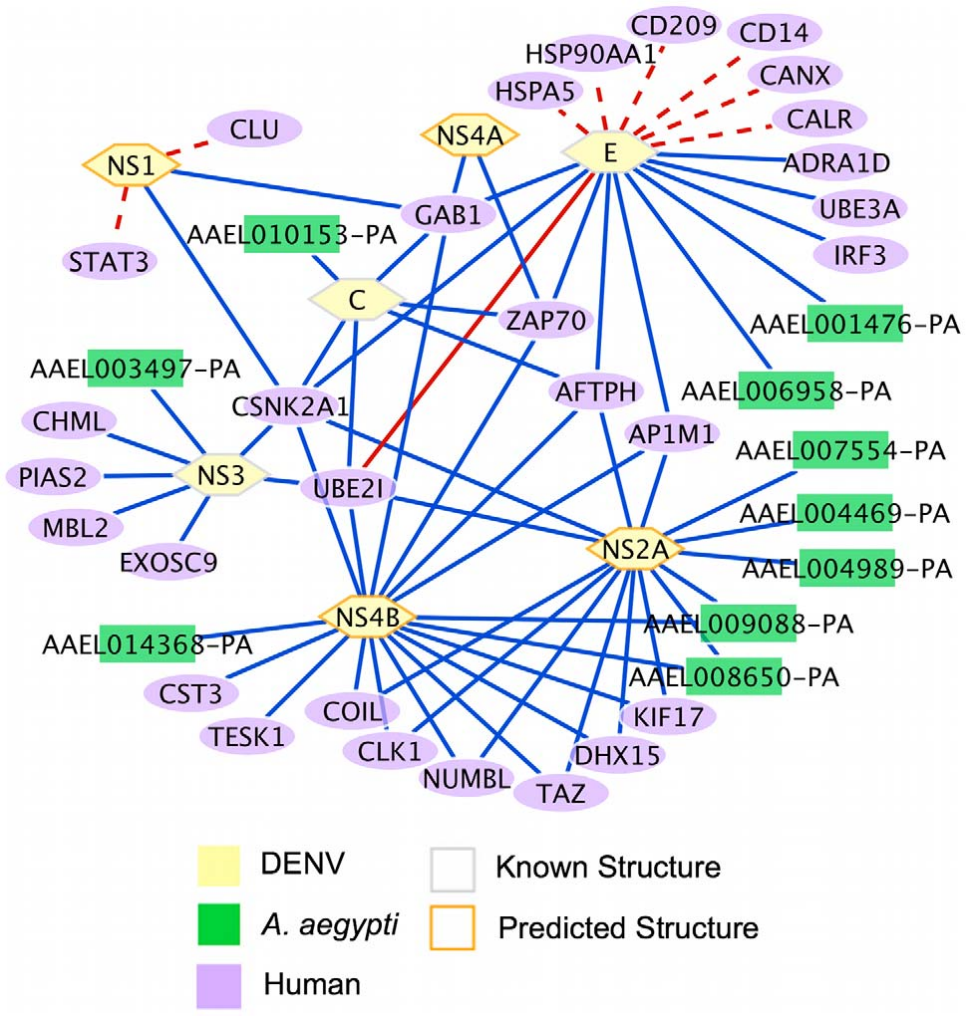
Predicted interactions with literature support. Predicted interactions between DENV and its hosts. Only predictions that were already known and those involving host factors are shown. Solid lines represent interactions for which the host protein was found by an siRNA screen to be involved in DENV infection, while dashed lines indicate that it is not a known host factor. Red lines represent interactions already known from the literature.

While host factors may act through mechanisms other than direct protein interactions, their functional involvement in DENV infection makes them more likely to participate in host-pathogen protein interactions. Comparing the identified host factors against our predictions, we found that 48 of the human target proteins were also identified as host factors in the siRNA screens, as well as 3 of the *D. melanogaster* targets (Figure 3 and Table 2). Many host proteins are predicted to interact with both NS2A and NS4B. This is due to apparent structural similarities between these two DENV proteins; several of the hDENV-similar proteins for NS2A and NS4B are the same. In particular, many predictions for both proteins are based on regions of structural similarity to members of the 14-3-3 family, protein phosphatase 2 regulatory subunits, and beta-catenin. These hDENV-similar proteins are key signaling proteins, with many known interactions. 14-3-3 proteins have been found to interact with over 200 polypeptides involved in highly diverse cellular functions [62] and PP2 is a serine-threonine phosphatase and tumor suppressor [63]. Beta-catenin is a well known member of the Wnt signaling pathway. The other two DENV proteins that have no known structure, and hence have predictions based on modeled structures, do not show structural similarity to 14-3-3 or protein phosphatase proteins. The combination of a predicted interaction along with siRNA functional relevance suggests that these host-DENV relationships would form the basis of a high-priority candidate list for future investigation.

#### Subcellular co-localization

As an additional method of identifying the most well-supported predictions, we also filtered predictions based on subcellular co-localization. The obvious assumption here is that for two proteins to directly interact, they must be physically present together within the same cellular compartment. Therefore, we used shared GO cellular component (CC) annotation to filter our predicted interactions. While this filtering should highlight a smaller set of proteins having additional evidence of interaction, we note that CC annotation is very noisy, with the localization of proteins within a cell being often poorly characterized for even well-studied model organisms, let alone species such as DENV or *A. aegypti*.

As GO annotations taken from DAVID for DENV are linked to entries for the DENV polyprotein rather than the individual proteins, all DENV proteins receive the same annotations. To ameliorate this problem and to include proteins with modeled structures, we assigned GO terms to the sequence corresponding to each DENV protein structure using a different tool, GOanna, which finds possible annotations based on the annotations of highly significant BLAST hits [36]. By assigning GO terms in this way, we were able to find CC terms for all of the DENV proteins.

**Figure 4:**
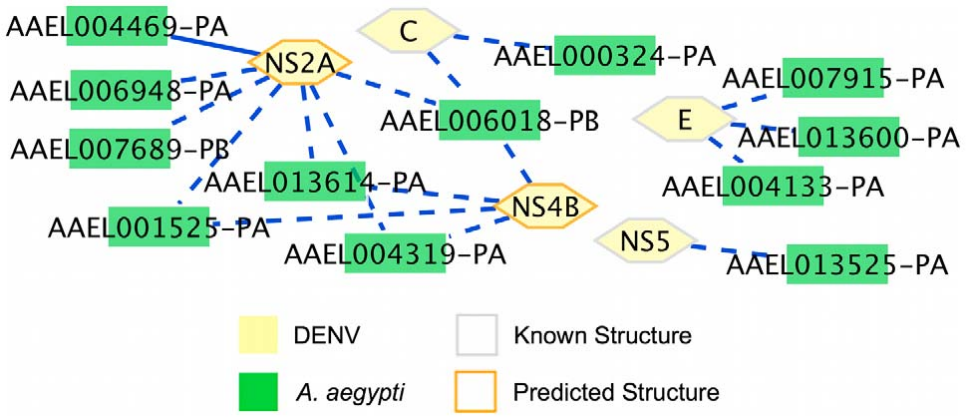
Predicted interactions in *A.aegypti* after CC filtering. Predicted interactions between DENV and *A.aegypti* where the DENV protein and its target share at least one GO CC term. Solid lines represent interactions for which the *A.aegypti* protein is a host factor.

We used this information to create a smaller list of predicted interactions, containing only those predictions where the DENV protein and the host target share at least one GO CC term. After CC filtering, there were 2,073 predicted interactions between DENV and 1,099 human proteins. Seven of the 19 known DENV-human interactions remained after filtering (reduced from 9 of 19), as well as 20 interactions involving host factors (Table 2). The two known interactions that were removed during CC filtering were UBE2I and HSP90AA1 interacting with E. The CC filter reduced both the total number of predictions and the ones containing host factors by about half, leaving many fewer predictions with only a small decrease in predictions already having known functional support. For the predicted interactions in *A. aegypti*, the reduction in predictions was more pronounced, with only 17 predictions and 12 targets passing the CC filter (Figure 4). One of these predictions involves a host factor, as compared to 12 of the 176 predictions before filtering. While GO compartment information is incomplete, CC filtering does provide a smaller list of predictions, and in the case of human-DENV interactions, with only a slight decrease in the ability to predict known interactions. Full tables of interactions incorporating CC filtering are provided in Tables S3 (human) and S4 (*A. aegypti*).

#### Prediction accuracy

It is difficult to judge the accuracy of predictions for protein interactions in host-pathogen systems, especially for those pathogens such as DENV that have received less attention than their worldwide burden deserves. However, our results include essentially half (47%) of the known interactions between DENV and human proteins, as well as a significant number of proteins from both human and fly that have been suggested to play a functional role in DENV infection [30]–[35]. Protein interaction prediction is a difficult problem, with even a slight improvement over random guessing considered a success. In a recent attempt to predict protein interactions in yeast, the organism with the most complete known interactome, the best method tested was able to predict 60% of the known interactions, outperforming several previous methods having accuracies as low as 38% [64]. Another study evaluating the performance of three prediction methods based on protein domains showed that all three performed only slightly better random guessing on benchmark datasets [65]. A large set of known interactions that could serve as a gold standard for humans and DENV is lacking, but based on the small number of DENV2-human interactions that are known, we estimate that the accuracy of our method is comparable to that of other methods applied to better characterized systems such as yeast. Certainly there are many protein-protein interactions and host factors that remain to be discovered. As previously mentioned, HIV, another virus which encodes only a handful of proteins, is known to participate in over 800 direct interactions with human proteins, with over 2500 interactions if ones that may be indirect are included [38]. It seems reasonable to assume that DENV2 also participates in a large number of host-pathogen interactions, most of which are currently unknown.

We have also applied this methodology to the prediction of protein interactions between HIV and human, a host-pathogen system for which much is known, although the same problems still exist to some extent [12]. In that work, we found that at the level of proteins, a minimum of 6% of predictions are correct. As there are often multiple protein/structure entries defined for each gene, when we consolidate proteins to single genes, at least 10% of our predictions were estimated to be correct when comparing predictions involving HIV host factors that passed CC filtering to known HIV-human protein interactions. This represented a significant improvement over random predictions (1% of random predictions were correct). For the DENV-host systems described in this work, we similarly estimate that, at a minimum, approximately 10% of predictions provided here are correct. It is encouraging that, comparable to yeast studies, we were able to find approximately 50% of the currently known interactions between DENV and human.

#### Comparison to another dataset

As discussed earlier, the major challenge in predicting protein interactions for pathogens is the lack of interaction data. A further complication is that, in addition to the lack of known host-pathogen interactions for DENV2, little is known about protein interactions within the mosquito vector itself. Very recently, a draft of the *A. aegypti* interactome was predicted by assuming that the mosquito orthologs of interacting proteins in *C. elegans*, *D. melanogaster*, and *S. cerevisiae* would also interact [13]. Since so little is known about the insect vector, we felt it would be valuable to compare our results to another set of predictions made using our general method, but based on this newly-predicted mosquito interactome. To do this, we found structural similarity between DENV2 proteins and fly, worm, or yeast proteins, mapped these similar proteins to their orthologs in mosquito, then used the mosquito interactome to predict host-pathogen interactions. In comparison, we previously used only fly proteins that were similar to DENV2 proteins, linked them to interacting proteins from experimentally determined fly interactions, then found the mosquito orthologs to map our predictions.

When we expanded our search for DENV-similar proteins to include all three model organisms, we found 45 proteins (vs. 15 in our original set) that showed structural similarities to DENV2 proteins. We then mapped these proteins to orthologs in *A. aegypti*. Any *A. aegypti* proteins predicted to interact with these orthologs, according to the recently published mosquito interactome, were predicted to interact with DENV2. As a result, we found 263 mosquito targets participating in 351 interactions with DENV2 proteins (Table S6). This can be compared to the 158 targets and 176 interaction predictions generated by considering *Drosophila* interactions alone (Table 2). The larger number of interactions predicted is a result of including more protein structures from additional species. Overall, the predictions based on the new data showed a similar GO term enrichment as compared to our original results (Figure S1).

It is difficult to directly assess prediction quality due to the absence of known interactions between DENV2 and mosquito. However, we note that despite the larger number of predictions, fewer involved proteins that were suggested to be host factors (10 compared to 12 in the original predictions). This suggests that the larger number of predictions may represent an increase in the number of false positive predictions. Indeed, some of the within mosquito interactions used to produce this larger set of interactions may also be false positives, as the interactions of [13] are predicted from interactions in other species. The evolutionary distances between *A. aegypti*, worm and yeast may be too great to provide accurate predictions of protein interactions using orthologs. A network of the fourteen predictions made using both datasets is given in Figure S2. In addition, Guo et al. predicted 22 interactions between DENV2 and *A. aegypti* by finding orthologs of proteins from any host species shown to interact with proteins from any flavivirus [13]. Only 3 of the *A. aegypti* proteins they predict to interact with DENV2 (AAEL012515, AAEL014959, and AAEL013600) are present among the predictions made with our method using *Drosophila* experimental interactions alone. In addition, we predict them to interact with different DENV2 proteins than what is predicted in [13].

### Stress and Apoptosis in DENV Pathogenesis

**Figure 5:**
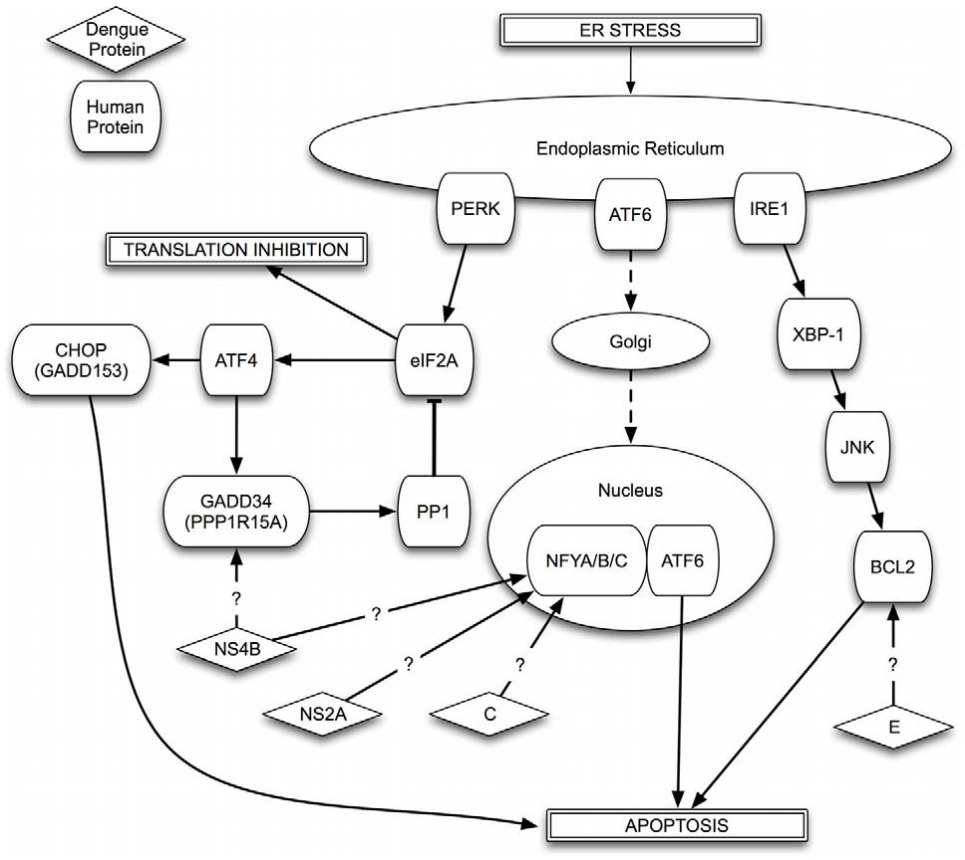
DENV and ER Stress. Potential interactions between DENV proteins and key components of the Unfolded Protein Response (UPR) and ER stress. See text for additional details.

We note several links between DENV pathogenesis, stress responses, and apoptosis among our predictions and in the literature. The GO term “regulation of stress response” is enriched among hDENV-similar proteins, as well as several terms related to apoptosis among the human target proteins. Several potential DENV receptors are involved in stress responses, such as HSP90, HSPA4, and HSPA5 [33], [41]. In particular, flaviviruses assemble within and bud from the ER, and are known to induce the Unfolded Protein Response (UPR), which reacts to stressors of ER function. As the UPR is necessary for cell survival during infection, but also has a negative impact on viral replication, modulation of this response by DENV may be advantageous. The UPR can induce either survival or apoptosis signals depending on the strength and duration of the ER stressor [66]. Three major branches, running through PERK, ATF6 and IRE1, regulate the UPR and ER homeostasis, and all three have been shown to be induced by DENV infection (Figure 5) [67].

Activation of PKR-like ER kinase (PERK), leads to phosphorylation of the eukaryotic initiation factor 2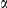 (eIF2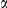). This phosphorylation inhibits the formation of translation initiation complexes, leading to translation inhibition and a reduction in the number of unfolded proteins within the ER [66]. Production of ATF4 is also enhanced as a result of eIF2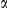 phosphorylation, eventually leading to GADD34 production. GADD34 acts within a negative feedback loop, recruiting protein phosphatase 1, leading to dephosphorylation eIF2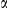 and the restoration of normal translation efficiency. Persistent ER stress leads to CHOP expression and promotion of apoptosis. It has been suggested that DENV may be able to compensate the UPR response by inducing dephosphorylation of eIF2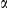 to restore translation [67]. We predict interactions between NS4B and GADD34 (PPP1R15A) (Figure 5). A listing of all predictions, with or without filtering, are provided in Tables S1, S2, S3, S4, S5.

ATF6 is a bZIP family transcription factor that transits from the ER to the Golgi in response to ER stress. It undergoes processing in the Golgi and transits to the nucleus, leading to upregulation of multiple apoptosis-relevant genes and eventual apoptosis. While we do not predict any direct interactions with ATF6, we do predict interactions between NS2A, NS4B, and C with associated pathway member NFYA, which forms a complex with ATF6 in response to ER stress [68] (Figure 5).

In the third branch of the UPR, the ER transmembrane protein IRE1, containing both kinase and RNase activities, becomes autophosphorylated and activated in response to ER stress, leading to XBP-1 splicing and translation of UPR relevant genes. Both DENV2 and Japanese Encephalitis Virus infection have been shown to activate XBP1 and its downstream genes in N18 mouse neuroblastoma cells, reducing the cytopathic effect of the virus [69]. Knockdown of XBP1 expression by siRNA has also been shown to lead to greater cytotoxicity in response to infection [69]. Persistent stress leads to apoptosis through an IRE-JNK-BCL2 pathway. Our predictions suggest potential interactions between E and BCL2. In addition, other BCL family members are also predicted to interact with DENV proteins including BCL2ll (BIM; a facilitator of apoptosis) with NS4B, BCL2L1 (BCLX; both pro- and anti-apoptotic splice variants) with E and NS3, and BCL2L10 (Boo; supression of apoptosis induced by BAX but not BAK) with NS3 (Figure 5).

A recent study investigating protein interactions between DENV envelope protein and host proteins described direct interactions between E and BiP (HSPA5), Calnexin (CANX) and Calreticulin (CALR) [55]. All three major ER stress transducers interact with BiP, which serves as a negative UPR regulator, and along with other ER chaperones, facilitates proper folding of proteins. Similarly, Calnexin and calreticulin are chaperones that bind to glycosylated proteins. Our methodology predicts each of these interactions with the E protein. In addition, we also predict that CALR is likely to interact with NS1 as well as NS3. Overall, these results suggests multiple sites within the host network at which DENV proteins can potentially manipulate the Unfolded Protein Response.

**Figure 6:**
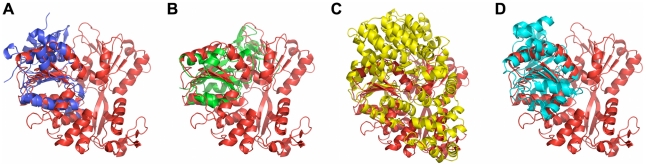
Structural similarities between NS3 and human proteins involved in apoptosis. (A) Structural similarity between NS3 and RAD51 (1n0wA [86]). (B) Structural similarity between NS3 and TK1 (1w4rA [87]). (C) Structural similarity between NS3 and APAF1 (1z6tC [88]). (D) Structural similarity between NS3 and DDX5 (3fe2A [89]). NS3 (2bhrA [90]) is shown in red.

In addition, several studies have implicated NS3 in DENV-induced apoptosis. The ability of DENV1 to cause apoptosis in HepG2 cells differs across strains. The mouse neurovirulent strain FGA/NA d1d differs from its parental strain, FGA/89 by 4 mutations, one of which leads to a non-conservative substitution in NS3. FGA/NA d1d was shown to have a reduced capacity to induce apoptosis, although whether this was mediated by the mutation in NS3 or by one of the other mutations, which were all in the E protein, is unclear [70]. However, Vero cells expressing DENV2 NS3 undergo apoptosis by a mechanism that is dependent on NS3 protease activity and enhanced by the presence of NS2B [71]. In addition, West Nile Virus NS3 is sufficient to induce caspase-8-depedent apoptosis, and is suggested to directly interact with, cleave, and activate caspase-8 in NIH 3T3 cells [72]. We predicted a number of interactions between NS3 and members of apoptotic pathways. A few examples of the structural similarities that led to these predictions are shown in Figure 6. NS3 was predicted to interact with p53 based on structural similarities with RAD51, TK1, and DDX5. Similarities with RAD51 also led to predicted interactions with ABL1, BRCA1/2, CASP3, and CASP7. Furthermore, NS3 has regions of similarity to APAF1, and is therefore predicted to interact with BCL2L1, BCL2L10, Fas, and the caspases -3, -4, -8, and -9. These results suggest that NS3 may play a role in DENV pathogenesis by influencing apoptosis in host cells, mediated by specific interactions between NS3 and host proteins.

### DENV and the Interferon Response

**Figure 7:**
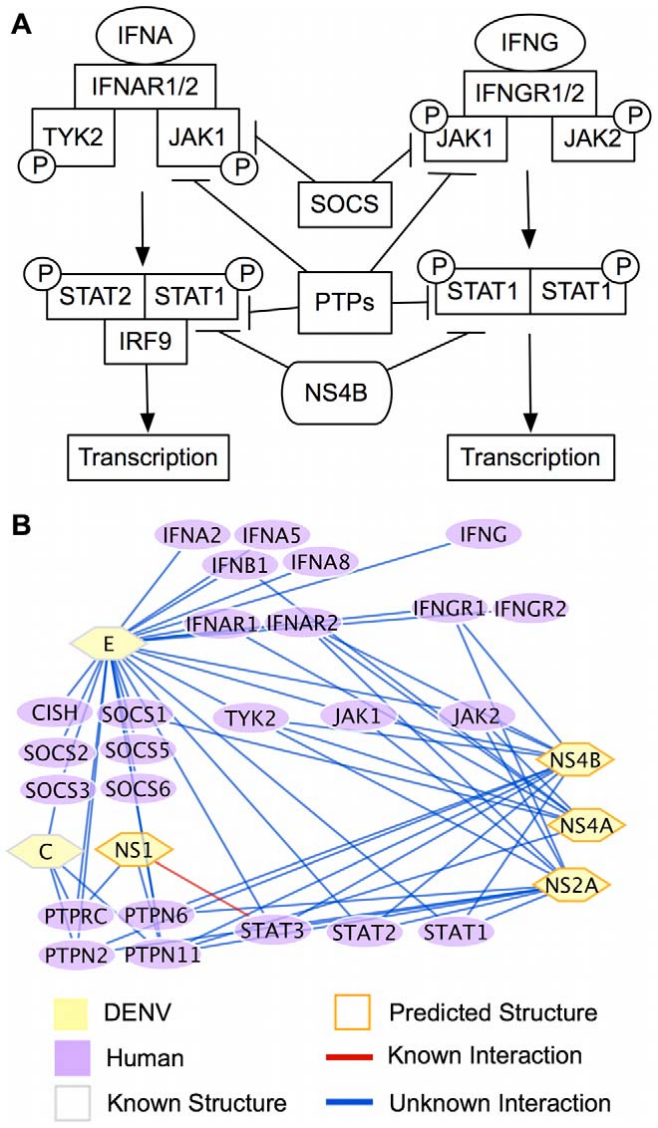
DENV influences IFN signalling. (A) Interferon signaling pathway. IFNA and IFNG bind to their respective receptors and cause the activation of Jak family tyrosine kinases. This activats of STAT proteins, which form hetero- or homodimers and induce the expression of IFN response genes. SOCS proteins negatively regulate JAK1 and PTP proteins negatively regulate JAK1 and STAT1. NS4B can reduce the phosphorylation of STAT1. (B) Predicted interactions between DENV proteins and members of the IFN-induced JAK-STAT pathway.

Humans and *A. aegypti* are known to use conserved defenses against DENV infection, involving several signaling pathways of the innate immune system, which is consistent with our finding of enriched GO terms related to the immune system among the target proteins of both humans and *A. aegypti*. In particular, the JAK-STAT signaling pathway has been shown to modulate susceptibility to DENV infection, in both mosquitos and humans [73], [74]. In humans, the JAK-STAT pathway can be activated by the interferons (IFN), IFN-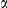, IFN-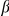, and IFN-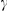, and mediates the antiviral response (Figure 7A). When IFN-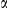 or IFN-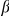 bind their receptor, IFNAR, the tyrosine kinases JAK1 and TYK2 are activated. This results in the phosphorylation and activation of STAT2 and STAT1, which then recruit IRF9 to form a transcription factor complex that transcribes IRF-7 and then the set of genes that are induced by IFN-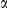. The interferon response is known to be induced upon DENV infection and high levels of IFN-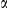 are normally present in the sera of DENV patients [75]. Furthermore, pretreatment of cells with IFN has been shown to block negative strand accumulation of DENV RNA, but this inhibition was strongly attenuated if treatment occurred even 4 hours after initial infection [76].

In fact, while infection induces an interferon response, several studies have shown that DENV interferes with the signaling pathway downstream of IFN-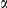. In dendritic cells, DENV is known to protect itself from the antiviral effects of IFN-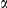 by reducing the phosphorylation of TYK2 and preventing the activation of STAT1 and STAT3, although the effects of IFN-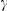 are not averted [74]. In addition, IFN-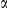-, but not IFN-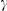-, dependent phosphorylation of STAT1 and STAT2 was found to be inhibited in A529 and HepG2 cells by the NGC strain of DENV2, but not by the strain TSV01, suggesting strain-dependent rather than serotype-specific differences in response [77]. Such strain-dependent differences also highlight the possibility of viral RNA sequence variations that potentially lead to changes in interaction specificity or the strength of interaction with host proteins.

A few specific proteins have been identified as modulators of the IFN response. For instance, IFN-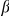 signaling was prevented by the viral proteins NS4B, and to a lesser degree by NS4A and NS2A [78]. Inhibition of signaling by NS4B was thought to occur through an observed reduction in the level of phosphorylated STAT1. Expression of STAT2 was also observed to be repressed following infection [79]. Recently, NS5 has also been shown to bind to STAT2, resulting in reduced IFN signaling [80]. In this same study, when expressed as a proteolytically processed precursor, NS5 was also found to target STAT2 for proteasome-mediated degradation. However, while it is clear that DENV is actively involved in modulating the host interferon response, there likely remain many specific interactions by which DENV proteins inhibit IFN signaling that are not known.

Our predictions suggest many potential interactions between DENV and multiple human proteins involved in the JAK-STAT pathway (Figure 7B). In particular, for NS4B, we have predicted possible interactions with IFNAR2, IFNGR1, JAK1, JAK2, TYK2, PTPN11, PTPN2, PTPN6, PKR (EIF2AK2), STAT1, STAT2, and STAT3. Thus, NS4B may reduce the observed phosphorylation of STAT1 through direct interactions with the host STAT1 protein, through interactions that effect the activities of proteins upstream of STAT1 (e.g. JAK1 or TYK2), or through interactions with at least one PTP protein, which are negative regulators of STAT activity. In addition, NS4B is predicted to interact with PKR, a key component of the IFN response in blocking virus replication. A close relative of DENV, hepatitis C virus, has been shown to inhibit interferon signaling through inhibition of PKR as well as by competing with eukaryotic translation initiation factor 2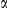 as a PKR substrate [81], [82]. NS2A is also predicted to interact with the same members of the this signaling pathway as NS4B. However, NS4A is predicted to interact with IFNAR1, IFNAR2, IFNB1, JAK2, TYK2, PTPN11, and SOCS1. In summary, we have predicted specific host-pathogen protein interactions that may enable DENV to escape the antiviral response induced by IFN and which can be tested in the future to determine the precise mechanism by which DENV manipulates this host system.

### Orthologous Human and *A. aegypti* Targets

**Figure 8:**
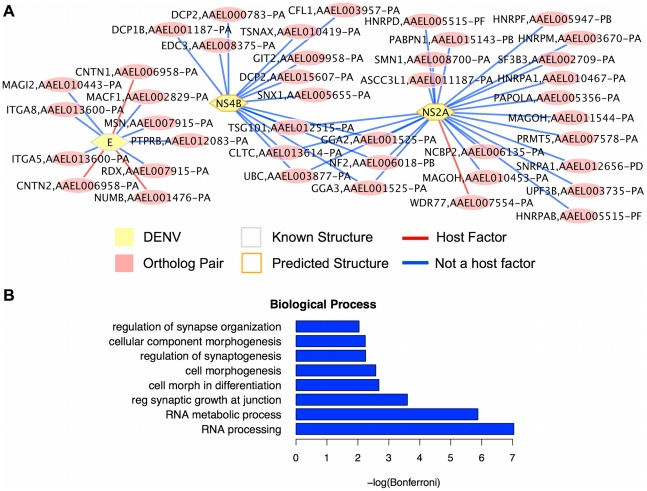
Predicted orthologous interactions. (A) Predicted interactions between DENV and orthologous pairs of *A.aegypti* and human proteins. The human protein is listed first, followed by its ortholog in *A.aegypti* which is also predicted to interact with the DENV protein. (B) GO biological process terms enriched among the interactions predicted to be conserved between human and *A.aegypti*. “reg synaptic growth at junction” is an abbreviation for “regulation of synaptic growth at neuromuscular junction,” and “cell morph in differentiation” stands for “cell morphogenesis involved in differentiation.”

To complete its lifecycle, DENV must survive in two very different hosts and must perform many of the same processes in each, such as transcription and translation. Since some proteins and essential processes are conserved between mosquitos and humans, it is possible that some of the proteins that are manipulated by DENV in one host are orthologous to the proteins used in the other host. To identify potential interactions of this type, we compared our interaction network predictions before CC filtering for human and *A. aegypti* to find proteins which are orthologous between the two hosts, as well as predicted to interact with the same DENV protein in each. We found 47 pairs of orthologs that were predicted to interact with the same DENV protein (Figure 8). Four of these predicted interactions represent host factor targets for *A. aegypti*. We note that our method depends on known interactions within species, and may miss some orthologous host-pathogen interactions if the within species interactions are not known.

To examine the functional role of these conserved interactions, we performed GO term enrichment for biological process and molecular function. No molecular function terms were significantly enriched (Bonferroni corrected p-value <0.01). Most of the biological processes represented by orthologous interactions in both hosts were also found enriched in the predictions for a single host. For example, at least 17 of the human proteins and their orthologs in *A. aegypti* are involved in mRNA processing or metabolism (ASCC3L1, DCP1B, DCP2, HNRPA1, HNRPD, HNRPF, HNRPM, UPF3B, WDR77, MAGOH, NCBP2, PABPN1, PAPOLA, PRMT5, SNRPA1, SF3B3, SMN1). Five of the interactions known to occur between DENV and human proteins involve the mRNA processing proteins HNRNPK, HNRNPC, PTBP1, and SSB [30], [47], [50], [53]. “RNA processing” and “RNA metabolic process” were highly enriched in the mosquito predictions and in the orthologous predictions.

In addition, we found a number of enriched GO terms relating to the regulation or formation of synapses among the predictions conserved in both species. Previously, the term “brain development” was enriched in the set of dDENV-similar proteins. DENV2 virus particles have been found in vesicles near the presynaptic membrane in spinal cords of SCID mice, and it was suggested that fusion of these vesicles at the synapse might aid the spread of DENV2 from neuron to neuron [83]. In *Culex pipiens quinquefasciatus* mosquitos, West Nile Virus, another flavivirus, was also found near synapses and in synaptic vesicles [84]. Furthermore, DENV3 is known to infect the nervous system of *A. aegypti*, altering the mosquito's feeding behavior by prolonging feeding and possibly enhancing the spread of DENV3 by making it more likely that feeding will be interrupted, and the mosquito will have to feed on additional humans [85].

The processes enriched in the predicted interactions conserved between the two hosts are consistent with the effects of DENV infection in each host. In particular, many of the terms enriched among the orthologous predictions are similar to terms enriched for mosquito predictions. This is not necessarily surprising, given that the mosquito prediction set is much smaller than the human one, but indicates that orthologous predictions in humans were made corresponding to many of the mosquito predictions. However, we found terms involving cell morphogenesis enriched among the orthologous predictions, but not within the predictions specific to either host. Therefore, the mosquito-specific predictions do not completely overlap with the human predictions, and new processes key to DENV infection in both hosts can be revealed.

### Conclusion

We have created a map of potential protein-protein interactions between the host-pathogen triad DENV2 and its hosts *H. sapiens* and *A. aegypti*. The computational methodology employed to generate this map assumes that proteins with comparable structures will share interaction partners. Therefore, we predict that DENV2 proteins may merge into the host protein interactome at the points normally occupied by structurally similar host proteins, creating an interface for the manipulation of downstream host processes. From previous studies, a number of human and fly proteins have been suggested to play some role in DENV2 infection, although the nature of this role is unknown in most cases. Using this methodology, we are able to make predictions regarding which host proteins may impact viral infection through interactions with specific DENV2 proteins. We note that the structural-based methodology here provides a larger picture of the interaction network, while more subtle changes at the sequence level are likely to explain experimentally observed differences in strain effects. Given the paucity of both structural and interaction data for this system, we cannot determine fine differences between strains, but this may be elucidated by further study. The networks presented here may help to provide a set of hypotheses for further investigation, potential therapeutic intervention, as well as help in improving our understanding of the DENV life cycle.

## Supporting Information

Figure S1 GO term enrichment of *A. aegypti* proteins based on data from Guo et al. [13]. (A) Enriched GO biological process terms. (B) Enriched GO molecular function terms. Light blue bars represent terms for *A. aegypti* targets, and pink is for terms from DENV-similar proteins. When more than ten terms were enriched for a set of proteins, only the ten most signicant terms are shown. Bonferroni corrected p-values were transformed by -log10. The following abbreviations are used: "translation factor nucleic acid bind" is "translation factor activity nucleic acid binding," and "macromolecular subunit organiz" is "macromolecular complex subunit organization."
(1.39 MB TIF)

Figure S2 Interactions predicted using both the original fly data and the mosquito interactome.
(0.51 MB TIF)

Table S1 Predicted interactions between DENV2 and human. Columns include the the DENV protein's Entrez Protein accession or PDB code, Uniprot accession, and name; the DENV-similar protein's PDB code, Uniprot accession, Entrez Gene ID, and Symbol; the target protein's Uniprot accession, Entrez Gene ID, and Symbol; if the target is a known host factor; and if the predicted interaction was already known in the literature.
(4.18 MB TXT)

Table S2 Predicted interactions between DENV2 and *A. aegypti*. Columns include the the DENV protein's Entrez Protein accession or PDB code, Uniprot accession, and name; the DENV-similar protein's PDB code, Uniprot accession, Entrez Gene ID, and Symbol; the *D. melanogaster* target protein's FlyBase gene number, and Symbol; the *A. aegypti* target protein's VectorBase protein ID, and Uniprot accession; and if the target is a known host factor.
(4.18 MB TXT)

Table S3 Predicted interactions between DENV2 and human after the CC Filter. Columns include the the DENV protein's Entrez Protein accession or PDB code, Uniprot accession, and name; the DENV-similar protein's PDB code, Uniprot accession, Entrez Gene ID, and Symbol; the target protein's Uniprot accession, Entrez Gene ID, and Symbol; if the target is a known host factor; and if the predicted interaction was already known in the literature.
(3.01 MB TXT)

Table S4 Predicted interactions between DENV2 and *A. aegypti* after the CC Filter. Columns include the the DENV protein's Entrez Protein accession or PDB code, Uniprot accession, and name; the DENV-similar protein's PDB code, Uniprot accession, Entrez Gene ID, and Symbol; the *D. melanogaster* target protein's FlyBase gene number, and Symbol; the *A. aegypti* target protein's VectorBase protein ID, and Uniprot accession; and if the target is a known host factor.
(0.01 MB TXT)

Table S5 Predictions involving orthologous human and *A. aegypti* target proteins. Columns include the the DENV protein's Entrez Protein accession or PDB code, and name; the *A. aegypti* target protein's VectorBase protein ID, Uniprot accession, and if it is a known host factor; the human target protein's Uniprot accession, Symbol, if it is a known host factor, and if the human interaction was already known in the literature.
(0.01 MB TXT)

Table S6 Predicted interactions between DENV2 and *A. aegypti*. Columns include the the DENV proteins Entrez Protein accession or PDB code, Uniprot accession, and name; the DENV-similar proteins PDB code, Uniprot accession, species, gene ID, and description; the *A. aegypti* ortholog proteins VectorBase pro- tein ID, geneID, and Uniprot; the *A. aegypti* target proteins VectorBase protein ID, gene ID, Uniprot accession, and description; and if the target is a known host factor or was also predicted by another group [13].
(0.04 MB TXT)
