# Supplementary figures and images for: Mapping Protein Interactions between Dengue Virus and Its Human and Insect Hosts
Source: PLoS Negl Trop Dis. 2011 Feb 15;5(2):e954. doi: 10.1371/journal.pntd.0000954 (PMC3039688; doi:10.1371/journal.pntd.0000954)

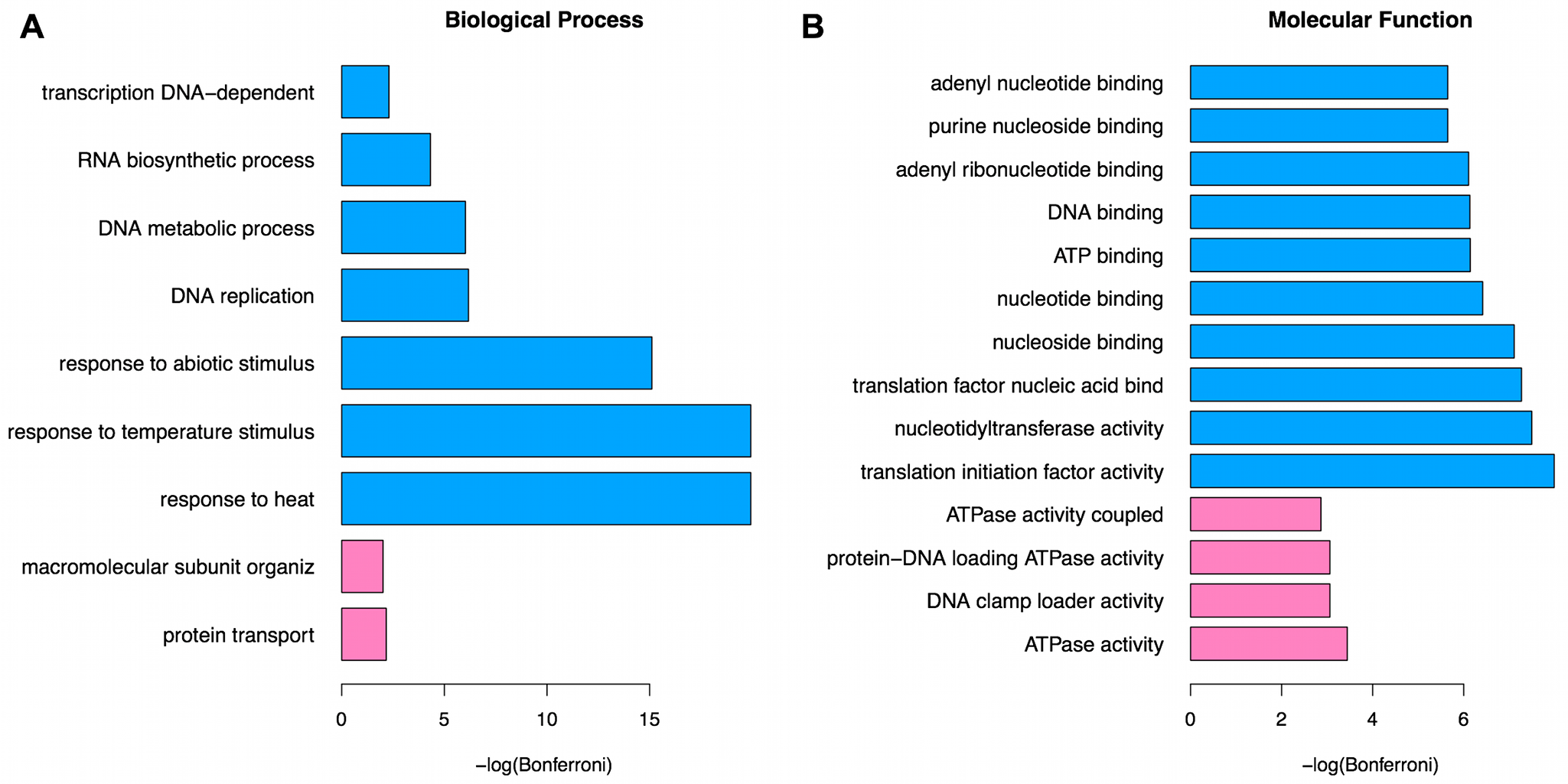

Supplement: Figure S1 — GO term enrichment of A. aegypti proteins based on data from Guo et al. [13]. (A) Enriched GO biological process terms. (B) Enriched GO molecular function terms. Light blue bars represent terms for A. aegypti targets, and pink is for terms from DENV-similar proteins. When more than ten terms were enriched for a set of proteins, only the ten most signicant terms are shown. Bonferroni corrected p-values were transformed by -log10. The following abbreviations are used: "translation factor nucleic acid bind" is "translation factor activity nucleic acid binding," and "macromolecular subunit organiz" is "macromolecular complex subunit organization." (1.39 MB TIF) [file pntd.0000954.s001.tif]

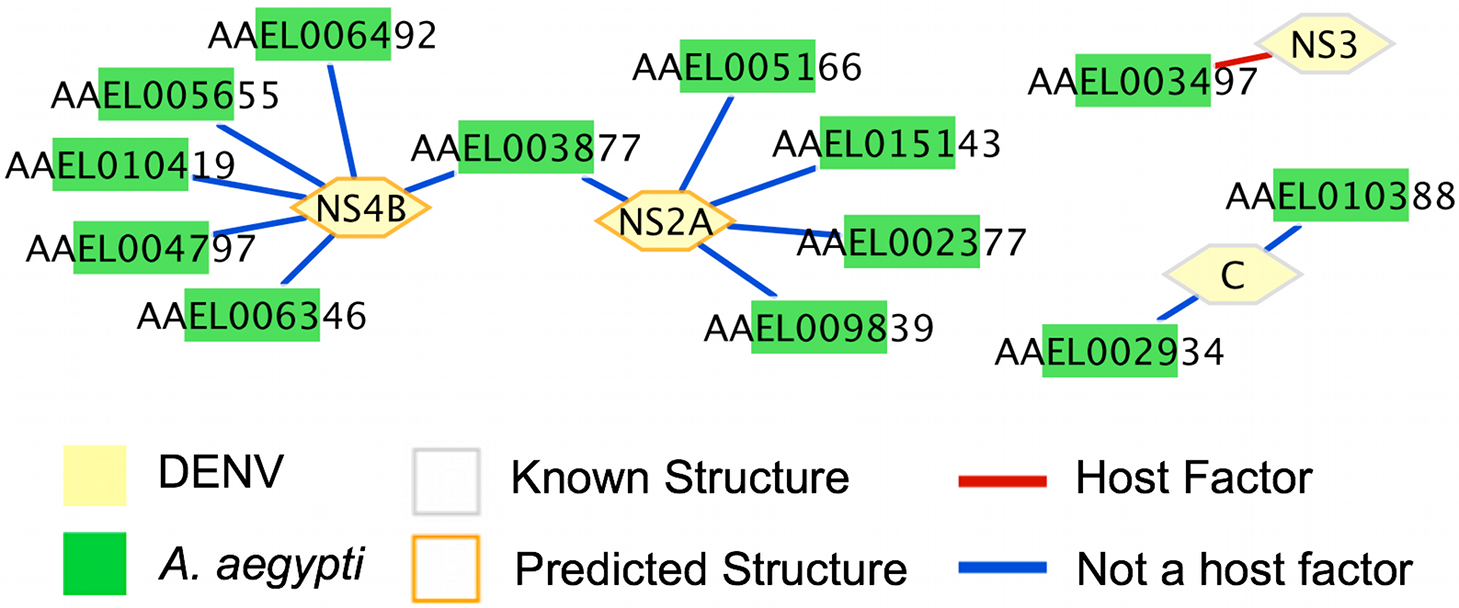

Supplement: Figure S2 — Interactions predicted using both the original fly data and the mosquito interactome. (0.51 MB TIF) [file pntd.0000954.s002.tif]
